# Chemical Characterization of Pruning Wood Extracts from Six Japanese Plum (*Prunus salicina* Lindl.) Cultivars and Their Antitumor Activity

**DOI:** 10.3390/molecules29163887

**Published:** 2024-08-16

**Authors:** Juan Ortega-Vidal, Nuria Mut-Salud, José M. de la Torre, Joaquín Altarejos, Sofía Salido

**Affiliations:** 1 Department of Inorganic and Organic Chemistry, Faculty of Experimental Sciences, Campus of International Excellence in Agri-Food (ceiA3), University of Jaén, 23071 Jaén, Spain; jov00007@red.ujaen.es (J.O.-V.); jmtorre@ujaen.es (J.M.d.l.T.); ssalido@ujaen.es (S.S.); 2 Institute of Biopathology and Regenerative Medicine, University of Granada, 18071 Granada, Spain; nmutsalud@gmail.com

**Keywords:** *Prunus salicina* (Rosaceae), pruning woods, agricultural wastes, phenolics, proanthocyanidins, HPLC quantification, total phenolic content, radical-scavenging activity, antitumor activity

## Abstract

The Japanese plum tree (*Prunus salicina* Lindl.) is mainly cultivated in temperate areas of China and some European countries. Certain amounts of wood (from pruning works) are generated every year from this crop of worldwide commercial significance. The main objective of this work was to value this agricultural woody residue, for which the chemical composition of pruning wood extracts from six Japanese plum cultivars was investigated, and the antiproliferative activity of extracts and pure phenolics present in those extracts was measured. For the chemical characterization, total phenolic content and DPPH radical-scavenging assays and HPLC‒DAD/ESI‒MS analyses were performed, with the procyanidin (−)-ent-epicatechin-(2α→*O*→7,4α→8)-epicatechin (**5**) and the propelargonidin (+)-epiafzelechin-(2β→*O*→7,4β→8)-epicatechin (**7**) being the major components of the wood extracts. Some quantitative differences were found among plum cultivars, and the content of proanthocyanidins ranged from 1.50 (cv. ‘Fortune’) to 4.44 (cv. ‘Showtime’) mg/g of dry wood. Regarding the antitumoral activity, eight wood extracts and four phenolic compounds were evaluated in MCF-7 cells after 48 h of induction, showing the wood extract from cv. ‘Songold’ and (‒)-annphenone (**3**), the best antiproliferative activity (IC_50_: 424 μg/mL and 405 μg/mL, respectively).

## 1. Introduction

Historically, the use of natural preparations, like infusions from plants or ointments, to alleviate symptoms produced by human diseases has been closely linked with society. Later on, a wide number of studies has reported their efficacy to treat several diseases and, hence, to play an important role in drug discovery. This is evidenced by the large number of known drugs with a natural origin, which proves the high interest about live organisms as inexhaustible sources of bioactive compounds [1,2]. Agricultural activity produces tons of waste around the world, some of them really interesting as pruning. Currently, this agricultural by-product shows some uses as biomass in order to produce energy, organic fertilizers after a fermentation process, and as vegetation cover to soil protection from erosion. In addition to the above, this cheap and valuable raw material could be a source of bioactive molecules as has been reported before [3]. Within this research topic, our group has shown some experience on valorisation of crops and pruning wood residues [4,5,6,7,8,9].

The Japanese plum (*Prunus salicina* Lindl., Rosaceae), notwithstanding the name, is considered to originate from China. However, *P. salicina* was introduced to the USA from Japan, adopting the above name [10]. Japanese plum crops are located in temperate areas, and also subtropical ones, showing a worldwide commercial significance. Fresh plum production in the world exceeded 12 million metric tons in 2021, according to FAO [11], although these statistics jointly show production data of European plum (*Prunus domestica* L.) and Japanese plum. China is the largest producer of plums in the world, well ahead of Romania, Serbia, Italy, and Spain, which are among the largest plum producers in Europe [12]. Numerous Japanese plum cultivars are currently known due to farming improvements, such as ‘Angeleno’, ‘Fortune’, ‘Red Beaut’, or ‘Friar’, amongst many others [10].

Plums are a highly nutritive fruit containing carbohydrates, fiber, minerals, vitamins, carotenoids, and phenolic compounds [13,14]. Among phenolic composition, there are hydroxycinnamic acid derivatives and flavonoids, such as anthocyanins, flavonols, flavanones, flavan-3-ols, and B-type procyanidins [15,16,17]. In addition, some authors have reported many healthy effects related to plum consumption, such as anticancer and anti-inflammatory activity and cardiovascular protective or antiallergic action [14,18,19]. Despite the high interest of fruit from this deciduous tree, the number of works focused on chemical composition from other organs, such as heartwood, bark, or leaves [20], is very limited.

Thus, the aims of this work were to carry out the chemical characterization of eight pruning wood samples of *Prunus salicina* Lindl., belonging to cultivars ‘Songold’, ‘Angeleno’, ‘Fortune’, ‘Red Beaut’, ‘Souvenir’, and ‘Showtime’ (by total phenolic content, antioxidant assays, HPLC–DAD, and HPLC–DAD/ESI–MS analyses), and to evaluate the antitumor activity of their extracts and some pure compounds by in vitro assays. This is the first time that the wood of the Japanese plum tree has been chemically studied and their extracts evaluated in terms of antiproliferative activity.

## 2. Results and Discussion

### 2.1. Sampling Collection and Extractions of Woods

Eight samples of pruning wood of Japanese plum (*P. salicina*) of six cultivars were collected on the same day during pruning works in Southern Spain (Table 1). Seven of these came from the experimental plots of *Centro IFAPA* “*Las Torres-Tomejil*”, Seville province, and another one from a farming plot located in Cordoba province. The extractions were carried out following an efficient method used previously in numerous occasions by us [6]. It consists of two extractions at reflux for 2 h, firstly, with dichloromethane (DCM) and, subsequently, with ethyl acetate (EtOAc). The purpose of the extraction with DCM is to eliminate non-polar compounds, without interest in working with phenolic antioxidants, prior to extraction with EtOAc. All samples showed yields with DCM (0.1–0.7%) lower than with EtOAc (0.6–1.5%) (Table 1). Focusing on EtOAc extracts, the cultivar ‘Showtime’ (1.5%, *Ps8*) showed the highest yield followed by ‘Red Beaut’ (1.2%, *Ps6*). These extraction percentages were around other pruning samples previously studied by us [6,8,21], but lower than a sample of European plum (*P. domestica* L.), cultivar ‘De la Rosa’ [7], which showed the highest value in our laboratory (3.1%).

### 2.2. Determination of Total Phenolic Content (TPC) and Antioxidant Activity of Prunus salicina Wood Ethyl Acetate Extracts

**Figure 1:**
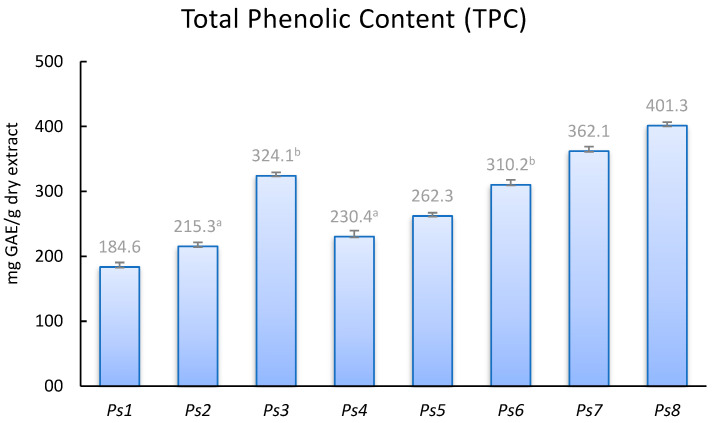
Total phenolic content (TPC) of *Prunus salicina* Lindl. wood EtOAc extracts (see Table 1 for cultivar names), expressed as the mean of three replicates. Error bars show the standard deviation (SD) of measurements; mean values not sharing the same letter are significantly different by ANOVA Tukey test (*p* < 0.05).

The evaluation of total phenolic content (TPC) and antioxidant activity of EtOAc extracts was carried out as a first approach to characterize the plum wood extracts. TPC was achieved using the Folin–Ciocalteu colorimetric methodology (Figure 1). The highest TPC value (456.5 mg GAE/g dry extract) corresponded to cv. ‘Showtime’ (*Ps8*) and the lowest one to cv. ‘Songold’ (*Ps1*). Focusing on the three samples of cv. ‘Angeleno’, the highest TPC number (324.1 mg GAE/g dry extract) was shown by a sample grown under organic management (*Ps3*), while *Ps2* and *Ps4*, collected from a conventional farm, showed similar values (215.3 and 230.4 mg GAE/g dry extract, respectively). In addition, these values were in the order of those found in woods of the European species (*Prunus domestica* L.) analysed by us [9]. Japanese plum woods also showed similar TPC compared to wood extracts obtained from apricot trees (*Prunus armeniaca* L.) [22]. However, our extracts reveal higher TCP content than stem and wood extracts from *Prunus avium* L. [23,24].

**Figure 2:**
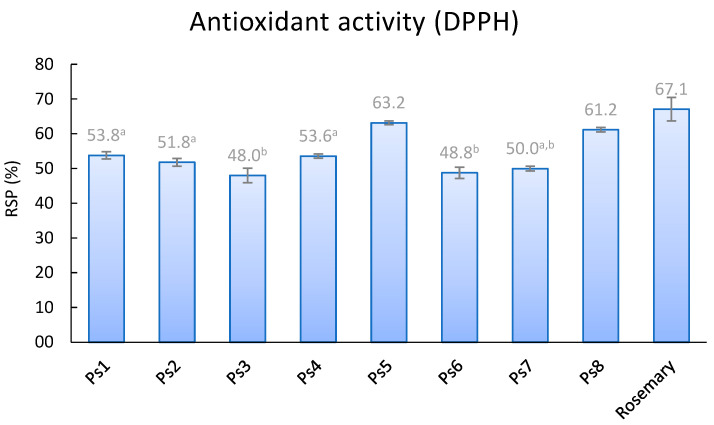
Radical-scavenging percentages (RSP) of *Prunus salicina* Lindl. wood EtOAc extracts (see Table 1 for cultivar names) and a commercial extract of rosemary, expressed as the mean of three replicates. Error bars show the standard deviation (SD) of measurements; mean values not sharing the same letter are significantly different by ANOVA Tukey test (*p* < 0.05).

The antioxidant activity of wood EtOAc extracts was determined by the widely used DPPH-scavenging assay. The radical scavenging percentages (RSP) values from ethyl acetate extracts ranged between 63.2% (*Ps5*) and 48.0% (*Ps3*) (Figure 2). The RSP results for the three samples of cv. ‘Angeleno’ (*Ps2*, *Ps3*, and *Ps4*) showed similar antioxidant activity. A sample of a commercial rosemary (*Rosmarinus officinalis* L.) extract was evaluated (67.1%) for comparison purposes, showing that all wood extracts had lower activities. Comparing with our previous work on *P. domestica* wood [9], Japanese plum samples were as antioxidant as European plum samples, except *P. domestica* cv. ‘De la Rosa’, which showed the highest percentage (72.9%) of all of them.

### 2.3. Identification of Components in Prunus salicina Wood Ethyl Acetate Extracts

**Figure 3:**
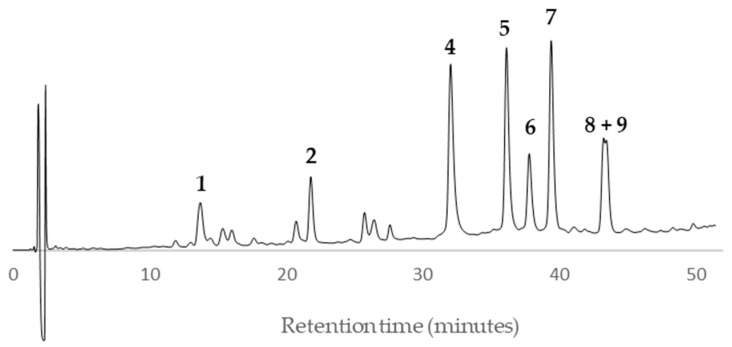
HPLC chromatogram of *Prunus salicina* Lindl cv. ‘Songold’ wood EtOAc extract (sample *Ps1*) at 230 nm. See Figure 4 for chemical structures and compound names.

Wood EtOAc extracts were analyzed by HPLC–DAD and HPLC–DAD/ESI–MS in order to know their phenolic profiles and to identify main components. Figure 3 contains the HPLC chromatogram of cultivar ‘Songold’ as representative of the general profile of the studied cultivars. The HPLC chromatograms of the rest of cultivars can be found in the Supplementary Materials file (Figures S1–S7).

**Figure 4:**
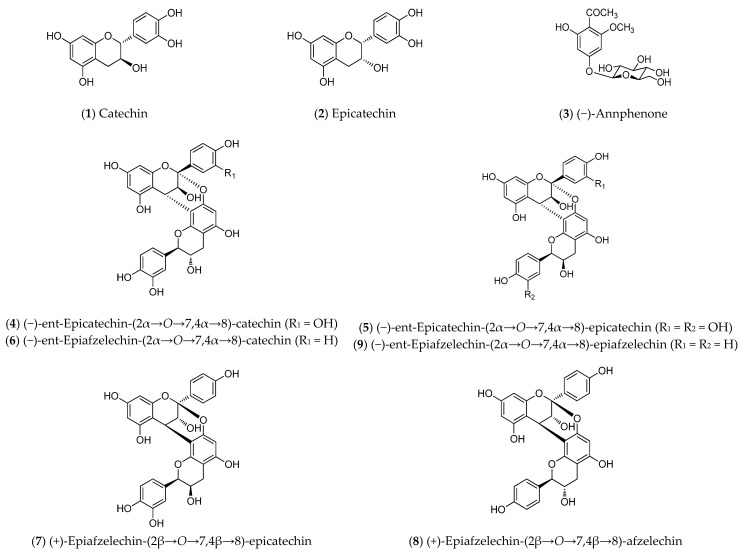
Compounds identified in wood EtOAc extracts of *Prunus salicina* Lindl. and previously isolated by us from a wood sample of *Prunus domestica* L. cv. ‘De la Rosa’ [7].

Peaks **1** and **2** showed a quasi-molecular ion peak [M − H]^−^ at *m*/*z* 288.6 (Table 2) according to the flavan-3-ol (epi)catechin, which was confirmed comparing their HPLC retention time (13.8 and 21.4, respectively) and UV spectrum with standards of (−)-catechin and (−)-epicatechin (Figure 4). The rest of the main peaks (**4**–**9**) showed a similar MS/MS fragmentation pattern, UV spectra, and HPLC retention times (Table 2) than compounds isolated and fully characterized by us from a wood EtOAc extract of European plum (*P. domestica* L.) cv. ‘De la Rosa’ [7]. It allowed us to identify two procyanidins (**4** and **5**, Figure 4) and four propelargonidins (**6**–**9**, Figure 4) in present Japanese plum wood samples. In addition, a low quantity of the phenolic glucoside (−)-annphenone (**3**) was identified in the studied extracts, which was isolated from *P. domestica* L. as well [7].

According to the HPLC profiles (Figure 3; Figures S1–S7), the major peaks in all extracts were **2**, **5**, **7**, **8**, and **9**. In addition, cultivar ‘Songold’ (*Ps1*) showed the peak **4** among the highest ones. On the other hand, a significant intensity of peak **1** only was detected in cultivars ‘Songold’ (*Ps1*), ‘Red Beaut’ (*Ps6*), ‘Souvenir’ (*Ps7*), and ‘Showtime’ (*Ps8*).

### 2.4. Quantification of Identified Compounds in Prunus salicina Wood Ethyl Acetate Extracts

The individual quantification of main components (**1**, **2**, **4**–**9**, Table 3) was achieved to complete the phytochemical characterization of EtOAc extracts. It was carried out using the external standard methodology. Commercial standards of the same chemical group were used to make calibrations curves. Thus, (−)-catechin was used to quantify flavan-3-ols (**1** and **2**) and procyanidin A-2 for proanthocyanidins (**4**–**9**). The cultivar ‘Showtime’ (*Ps8*) showed the highest concentration in both compound families, flavan-3-ols (1.37 mg per g of DW) and proanthocyanidins (4.44 mg per g of DW). The second highest concentrations found of flavan-3-ols and proanthocyanidins were in cv. ‘Souvenir’ (*Ps7*) and ‘Red Beaut’ (*Ps6*), respectively. Conversely, cv. ‘Fortune’ showed the lowest total concentration in polyphenols (1.74 mg per g of DW). The content of epicatechin (**2**) was upper than that of catechin (**1**) in all samples. Focusing on proanthocyanidins, compounds **5** and **7** were among of the most abundant in all of them, and **4** also in cv. ‘Songold’ (*Ps1*) and cv. ‘Souvenir’ (*Ps7*) samples. Concerning samples of cv. ‘Angeleno’ from different localizations, *Ps2* and *Ps3* showed similar concentration of flavan-3-ols (0.43 and 0.45 mg per g of DW, respectively) and lower than *Ps4* (0.68 mg per g of DW). Concerning the concentration of proanthocyanidins in ‘Angeleno’ samples, the highest value was found in *Ps4* (2.81 mg per g of DW), followed by *Ps2* (2.55 mg per g of DW), and the lowest quantity was in *Ps3* (1.91 mg per g of DW), grown under organic management. Therefore, although (−)-annphenone (**3**) was detected by HPLC–DAD/ESI–MS in some extracts, it was not quantified due to the low concentration.

According to this analysis, the wood of *Prunus salicina* Lindl. is rich in dimeric A-type PACs, in opposition to fruits that mainly contain B-type PACs according to other authors [16,17]. Hence, this agricultural waste seems to be an interesting source of A-type PACs, which shows upper concentrations than other natural sources like wood of some Pinus species (0.90–1.16 mg/g) [25] or almond skin [26], although slightly lower than *Prunus domestica* L. cv. ‘De la Rosa’ wood (8.51 mg/g) [7] or peanut skin (6.28 mg/g) [27]. Furthermore, it is remarkable that A-type PACs natural sources are less common than B-type PACs sources [28], suggesting that Japanese plum pruning wood is of particular importance as a raw material for this family of compounds.

To the best of our knowledge, this is the unique study reported on phenolic composition of *Prunus salicina* L. wood. Thus, this work showed a significant interest due to the healthy effect of proanthocyanidins, specifically those with a A-type link, which have special importance in the prevention of urinary tract infections [29]. Other authors have also been reported beneficial properties against virus infections [30], anti-inflammatory activity, and cancer, among others [31].

### 2.5. Antiproliferative Activity of Prunus salicina Wood Ethyl Acetate Extracts and Components

On this occasion, we were interested in knowing the antitumor activity of the pruning wood remains of the Japanese plum tree. For that, the antiproliferative activities of eight Japanese plum wood extracts (*Ps1*–*Ps8*) together with that of four pure compounds present in those extracts (**3**–**6**) were evaluated in MCF-7 cells after 48 h of induction (Table 4). Both extracts and compounds seemed to inhibit cellular proliferation in a dose–response manner and just two of the compounds (**4**, **6**) did not affect the viability of tumor cells at 200 μg/mL. The most effective extracts at 200 μg/mL were *Ps6* and *Ps7*, which decreased the cell population by 39.4% and 40.1%, respectively, followed by *Ps1* (30.2%). The best antitumoral activities of pure compounds at 200 μg/mL corresponded to **3** and **5** (28.8% and 19.2% of reduction, respectively). Based on these values, it can be deduced that pure compounds separately have less activity in general than when they are all together as part of an extract, with the exception of extracts *Ps2* and *Ps3*. It means that some synergistic effect among pure compounds should be operating. Focusing our attention on the most active extracts, samples *Ps7* and *Ps6* have in common a high proportion of A-type PACs (Table 3), which could point to, in a first approximation, that compounds **4**–**9** are behind the antiproliferative activity of the extracts. However, extract *Ps8* has the highest amount of A-type PACs of all extracts (Table 3) and had lower activity than, for instance, extract *Ps1*, with the half of A-type PACs (Table 3). Regarding the IC_50_ parameter, the lowest values corresponded to compound **3** and extract *Ps1* (Table 4). (−)-Annphenone (**3**) is a minor component in all extracts (Table 3) and a relationship between its presence and the activity of a given extract cannot be established. Therefore, the relationship between chemical composition of extracts and antitumor activity is not clear to us and additional work should be done in order to clarify this issue.

Regarding Japanese plums (fruits), a study reported in the literature showed that plum extracts, with a phenolic content different to that described here for pruning woods, were able to reduce the cell viability of two types of breast tumor cells, including MCF-7, and their effects were also concentration-dependent [32].

## 3. Materials and Methods

### 3.1. Chemicals

Solvents as dichloromethane (DCM) and ethyl acetate (EtOAc) used for extractions, and acetonitrile (ACN) for high-performance liquid chromatography (HPLC) analyses, were purchased from VWR (Barcelona, Spain). Ultrapure water used for HPLC analyses was produced by a Milli-Q-Water (1.8 MΩ) equipment (Merck, KGaA, Darmstadt, Germany). (−)-Catechin (99% of purity by HPLC) used for quantification purposes was isolated from *Prunus avium* L. wood [6]. Procyanidin A-2 (98% of purity by HPLC) used as standard was purchased from Extrasynthese (Genay, France). 2,2-Diphenyl-1-picrylhydrazyl radical (DPPH) used for radical-scavenging activity was purchased from Sigma-Aldrich Chemie (Steinheim, Germany). Rosemary extract used as reference was purchased from Evesa (Cádiz, Spain). Folin-Ciocalteau reagent used in total phenolic content determination was purchased from Merck Chemicals (Darmsdadt, Germany).

### 3.2. Plant Material Collection and Extraction

**Scheme 1:**
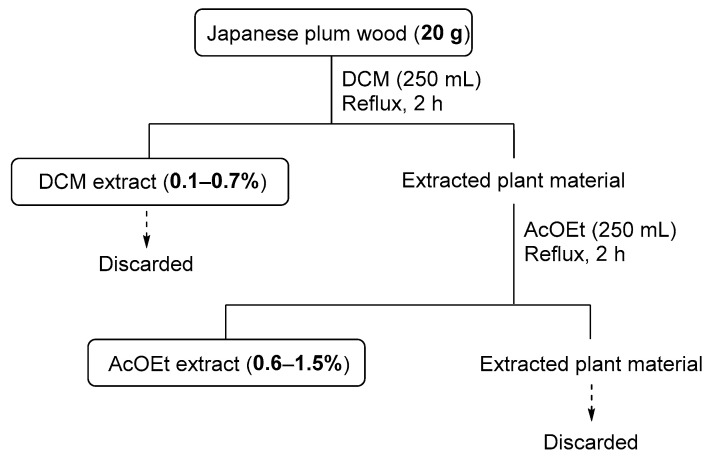
Extraction procedure of *Prunus salicina* Lindl. wood samples.

Eight samples (*Ps1*–*Ps8*) of pruning wood from six *P. salicina* Lindl. cultivars (‘Songold’, ‘Angeleno’, ‘Fortune’, ‘Red Beaut’, ‘Souvenir’ and ‘Showtime’) were collected in October 2015 during the pruning works of this fruit tree in the *Centro IFAPA* “*Las Torres-Tomejil*”, Alcalá del Río, province of Seville, Spain (N: 37°30′48″ W: 5°57′46″), and in a farming plot (“*Finca La Veguilla*”; N: 37°49′14″ W: 4°54′12″) located in Gualdalcázar, province of Córdoba, Spain. Both farms are located in the Guadalquivir River Valley, Southern Spain. The plant collection in *Centro IFAPA* was carried out in two experimental plots of Japanese plum trees with similar physicochemical soil composition and located 200 m apart one to another to avoid interference between the different pesticide treatments [33]. One of the plots was managed under conventional agriculture receiving applications of mineral manures, including complex formulations, ammonium nitrate and potassium sulfate. The other plot was under organic agriculture management and had cover greens (beans, oat, vetch), being fertilized once per year with just animal manure. From the conventional plot were collected the six cultivars while from the organic plot was only collected cultivar ‘Angeleno’ [33]. Regarding plant collection in “*Finca La Veguilla*” it was carried out in a conventional production plot of Japanese plum trees where just cultivar ‘Angeleno’ was picked up for comparison purposes. Collection works were supervised by Dr. Francisco T. Arroyo (Researcher at the *Centro IFAPA* “*Las Torres-Tomejil*”, Spain, and expert person in charge of supervising Japanese plum plantations and advising farmers in the area [34,35,36]) who confirmed the identify and origin of each wood sample. For plant material collection three trees of each cultivar were randomly chosen from which around ten non-productive shoots were cut in all cardinal directions of the tree. Thus, plant material consisted in pieces of leafless wood (with a diameter of 0.8–1.4 cm) that were dried at room temperature for 3 months in a dry and dark place with passive ventilation and chipped before using. Chopped samples were extracted with DCM and, then, with EtOAc (Scheme 1). In short, chopped woods (20 g) were extracted successively with dichloromethane (DCM) and ethyl acetate (EtOAc) (250 mL of each) for 2 h at reflux under nitrogen atmosphere, following a protocol previously optimized by the authors [6]. Solvents were evaporated under reduced pressure to give the corresponding DCM and EtOAc extracts (i.e., dry extracts). DCM dry extracts were discarded, whereas all EtOAc dry extracts were stored under argon in sealed vials at −20 °C until analysis.

### 3.3. Total Phenolic Content (TPC) and Antioxidant Activity

The total phenolic content (TPC) value of each EtOAc extract was determined by the Folin-Ciocalteu colorimetric method [37] using gallic acid as standard. The calibration curve (y = 0.0128x − 0.0794, r^2^ = 0.9987) was performed recording absorbances at 760 nm from methanolic solutions (20–75 µg/mL) of gallic acid on a Varian Cary 4000 spectrophotometer (Varian Inc., Palo Alto, CA, USA) [9]. Briefly, 0.6 mL of an aqueous solution of Folin-Ciocalteu reagent (0.2 N), and 0.12 mL of methanolic solutions of each ethyl acetate extracts (at 10 μg/mL) were added to a 1-cm path length semi-micro cuvette. The mixture was incubated in the dark for 5 min at room temperature. Next, 0.48 mL of an aqueous solution of Na_2_CO_3_ (7.5 g/L) was added, shaken, and incubated again for 1 h in the dark at room temperature. Finally, the absorbance at 760 nm was determined. Results were obtained as mg of Gallic acid equivalent (GAE) per gram of dry extract. Three replicates of each sample were measured.

The antioxidant activity of each EtOAc wood extract was achieved by the DPPH radical-scavenging method [9]. Briefly, 0.4 mL of methanolic solutions (50 µg mL^−1^) of EtOAc extracts were mixed with 0.8 mL of a methanolic solution of DPPH radical (7.09 × 10^−5^ M) in semi-micro cuvettes, shaken and kept in dark for 15 min at room temperature. In the case of blank, the mixture was prepared with 0.8 mL of DPPH radical and 0.4 mL of MeOH. Next, the absorbance was measured at 515 nm on a Varian Cary 4000 spectrophotometer. A calibration curve (y = 0.1114x + 0.020, r^2^ = 0.9919) was used to determine the concentration of DPPH radical in each solution. Three replicates of each sample were measured.

Antioxidant activity results were expressed as radical-scavenging percentage (RSP) and were determined using the following formula [9]:

$$RSP=\left[\frac{A_{B}-A_{A}}{A_{B}}\right]\times 100$$

where A_B_ is the absorbance of the blank (t = 0 min) and A_A_ is the absorbance of the evaluated solution (t = 15 min).

### 3.4. Analyses by HPLC–DAD and HPLC–DAD/ESI–MS of EtOAc Extracts

All EtOAc extracts (*Ps1*–*Ps8*) were analysed by HPLC–DAD using the same instrument described by us [6]. The mobile phase consisted in mixtures of ACN (solvent A) and H_2_O (solvent B), both with 0.2% AcOH, at a flow of 0.7 mL/min. The optimal separation was achieved with the following gradient method: 0.0–40.0 min, 5–20% A; 40.0–45.0 min, 20–25% A; 45.0–60.0 min, 25–40% A; 60.0–62.0 min, 40–5% A. Ethyl acetate extracts were analysed by HPLC–DAD/ESI–MS, using an instrument and analysis conditions described previously by us [7]. The mobile phase consisted in mixtures of ACN (solvent A) and H_2_O (solvent B), both with 0.2% AcOH, at a flow of 0.25 mL/min. The optimal separation was achieved with the following gradient method: 0.0–22.5 min, 5–20% A; 22.5–27.5 min, 20–25% A; 27.5–32.5 min, 25–40% A; 32.5–33.5 min, 40–5% A. MS parameters were described before by us [5].

### 3.5. Quantification of Phenolics Compounds in EtOAc Extracts

The quantification of identified compounds in EtOAc extracts was achieved by an external standard method using their HPLC peak areas at 230 nm as previously reported by us [7]. Calibrations curves were made using solutions (0.1–1 mg/mL in methanol) of (−)-catechin [6] and a commercial standard of procyanidin A-2, and correlating the area value of each peak with its concentration [7]. The concentration of each compound was expressed as milligrams of compound per gram of dry wood (DW).

### 3.6. Cell Lines and Culture

The human breast adenocarcinoma line MCF-7 (ECACC 86012803) was obtained from Cell Cultures Unit of the University of Granada (Spain). Cells were cultured at 37 °C and 5% CO_2_ with humidified atmosphere, using DMEM supplemented with 10% FBS, 10 mL/L penicillin-streptomycin 100×, and 2 mM L-glutamine.

### 3.7. In Vitro Antiproliferative Assays

MCF-7 cells were seeded in sterile 96-well plates (Thermo Fisher Scientific, Roskilde, Denmark) at high density (1.5 × 10^4^ cells/well) and incubated for 24 h to allow cell adhesion. All extracts were first dissolved in DMSO. From these solutions, different dilutions to be tested were prepared using Dulbecco’s Modified Eagle Medium (DMEM) without FBS (fetal bovine serum) or antibiotics. When the extract or pure compound solutions were added to cells for the induction, the DMSO concentration remained below 0.1% (*v*/*v*). Increasing concentrations of all extracts and compounds were added in the corresponding wells and incubated for 48 h. The effect on cells viability was evaluated using a colorimetric technique with sulforhodamine-B (SRB) [38]. Optical density values were determined in a microplate reader (Multiskan EX, Thermo Electron Corporation (Waltham, MA, USA)) at 450 nm. The assessment of absorbance was obtained using “SkanIt” RE 5.0 for Windows v. 2.6 (Thermo Labsystems, Waltham, MA, USA) and a regression analysis was carried out with the Statgraphics (Centurion 15) software (Statistical Graphics Corp, 2000, The Plains, VA, USA). The IC_50_ values were calculated from the semi-logarithmic dose–response curve by linear interpolation. All assays were performed in duplicate.

### 3.8. Statistical Analysis

The results are expressed as mean values ± SD. A statistical analysis of the data was carried out using GraphPad Prism version 5.00 for Windows (GraphPad Software, La Jolla, CA, USA). Statistical significative differences among measurements were determined by a one-way analysis of variance (ANOVA) with Tukey’s test (*p* < 0.05).

## 4. Conclusions

Pruning wood of Japanese plum tree (*Prunus salicina* Lindl.) could be an excellent natural source of A-type proanthocyanidins, the cultivar ‘Showtime’ showing the higher amount among all studied samples. The characterization of the wood extracts of six plum cultivars was performed by total phenolic content and DPPH radical-scavenging assays and by HPLC–DAD and HPLC–DAD/ESI–MS analyses. It allowed for the identification of flavan-3-ols catechin (**1**) and epicatechin (**2**), the phenolic glucoside annphenone (**3**), and six A-type proanthocyanidins (**4**–**9**). After quantification of components by an external standard method, proanthocyanidins **5** and **7** were the major components in most of the plum cultivars. Regarding the antiproliferative activity in MCF-7 breast tumor cells activity of both wood extracts and pure compounds **3**–**6**, most of the samples evaluated were able to achieve a reduction of up to 40% in the cell population with concentrations of 200 μg/mL. These results highlight the potential use of pruning wood from *P. salicina* as a renewable, cheap, and abundant source of bioactive compounds.

## Figures and Tables

**Table 1:** *Prunus salicina* Lindl wood samples studied in this work and extraction yields.

Pruning Samples		Extraction Yields ^a^	
Cultivar	Reference	DCM	EtOAc
‘Songold’ ^b^	*Ps1*	0.1	0.9
‘Angeleno’ ^b^	*Ps2*	0.4	0.9
‘Angeleno’ ^b,c^	*Ps3*	0.3	0.7
‘Angeleno’ ^d^	*Ps4*	0.5	1.0
‘Fortune’ ^b^	*Ps5*	0.3	0.6
‘Red Beaut’ ^b^	*Ps6*	0.4	1.2
‘Souvenir’ ^b^	*Ps7*	0.7	1.1
‘Showtime’ ^b^	*Ps8*	0.4	1.5

^a^ Result expressed as a percentage (grams of extract per 100 g of dry wood); ^b^ Sample collected in *Centro IFAPA* “*Las Torres-Tomejil*”, Seville, Spain; ^c^ Sample grown under organic management; ^d^ Sample collected in “*Finca La Veguilla*”, Cordoba, Spain.

**Table 2:** Chromatographic and spectral characteristics of the main HPLC peaks from wood EtOAc extracts of *Prunus salicina* Lindl.

Peak	t_R_ (min)	λ_max_ (nm)	[M − H]^−^
**1**	13.8	279.3	288.6
**2**	21.4	279.3	288.6
**3**	24.3	285.2	342.7
**4**	31.2	278.1	574.7
**5**	35.2	276.9	574.7
**6**	36.6	278.1	558.7
**7**	38.2	276.9	558.7
**8** and **9**	42.1	272.2	542.7

**Table 3:** Quantification of phenolic compounds identified in wood EtOAc extracts of *Prunus salicina* Lindl.

Compounds	Concentration of Components (Milligrams of Compound Per Gram of DW) ^#^							
	*Ps1*	*Ps2*	*Ps3*	*Ps4*	*Ps5*	*Ps6*	*Ps7*	*Ps8*
*Flavan-3-ols*								
**1**	0.21 ± 0.01 ^a^	0.10 ± 0.01 ^b^	0.11 ± 0.01 ^b^	0.16 ± 0.01	0.06 ± 0.01	0.22 ± 0.01 ^a^	0.31 ± 0.01	0.51 ± 0.01
**2**	0.22 ± 0.02 ^a^	0.33 ± 0.01 ^b^	0.34 ± 0.01 ^b^	0.52 ± 0.01 ^c^	0.18 ± 0.01 ^a^	0.43 ± 0.01	0.51 ± 0.01 ^c^	0.86 ± 0.05
Total *	*0.43*	*0.43*	*0.45*	*0.68*	*0.24*	*0.65*	*0.82*	*1.37*
**3** ^&^	<LOQ ^&^	<LOQ ^&^	<LOQ ^&^	<LOQ ^&^	<LOQ ^&^	<LOQ ^&^	<LOQ ^&^	<LOQ ^&^
*Proanthocyanidins*								
**4**	0.60 ± 0.05 ^a^	0.18 ± 0.01 ^b^	0.18 ± 0.02 ^b^	0.22 ± 0.01 ^b^	0.18 ± 0.04 ^b^	0.42 ± 0.01	0.56 ± 0.01 ^a^	1.02 ± 0.04
**5**	0.58 ± 0.02 ^a^	0.56 ± 0.03 ^a,b^	0.64 ± 0.04 ^a^	0.90 ± 0.01 ^c^	0.48 ± 0.02 ^b^	0.92 ± 0.03 ^c^	0.86 ± 0.03 ^c^	1.57 ± 0.04
**6**	0.25 ± 0.01 ^a^	0.13 ± 0.01 ^b^	0.14 ± 0.01 ^b^	0.17 ± 0.01	0.12 ± 0.01 ^b^	0.24 ± 0.01 ^a^	0.24 ± 0.01 ^a^	0.34 ± 0.01
**7**	0.53 ± 0.02 ^a^	0.97 ± 0.04 ^b^	0.56 ± 0.05 ^a^	0.98 ± 0.03 ^b^	0.47 ± 0.04 ^a^	0.97 ± 0.03 ^b^	1.11 ± 0.07 ^c^	1.12 ± 0.04 ^c^
**8** and **9**	0.42 ± 0.01 ^a^	0.71 ± 0.03	0.39 ± 0.02 ^a^	0.54 ± 0.01	0.25 ± 0.01	0.81 ± 0.04	0.38 ± 0.01 ^a^	0.41 ± 0.01 ^a^
Total **	*2.38*	*2.55*	*1.91*	*2.81*	*1.50*	*3.36*	*3.15*	*4.44*

DW: Dry wood; ^#^ values (mg/g DW) are mean ± SD of three replicates. * Sum of **1** and **2**. ^&^ Concentration of compound **3** was lower than the limit of quantification (LOQ). ** Sum of **4**–**9**. Mean values in the same line (row) not sharing the same letter are significantly different by ANOVA Tukey test (*p* < 0.05).

**Table 4:** Antiproliferative activity of wood EtOAc extracts of *Prunus salicina* L. and some pure compounds in MCF-7 cells.

Extract/Compound	% Reduction at 200 μg/mL	IC_50_ (μg/mL) *
*Ps1*	30.2 ^a^	423.8 ± 8.3
*Ps2*	8.9	768.9 ± 16.8
*Ps3*	10.1	789.3 ± 17.5
*Ps4*	24.2	471.2 ± 8.8
*Ps5*	29.8 ^a^	450.5 ± 10.2
*Ps6*	39.4 ^b^	518.2 ± 13.7
*Ps7*	41.0 ^b^	500.6 ± 12.5
*Ps8*	20.8	566.7 ± 12.3
**3**	28.8 ^a^	404.7 ± 7.4
**4**	Non affected	833.3 ± 16.6
**5**	19.2	461.9 ± 9.7
**6**	Non affected	882.3 ± 20.3

* IC_50_ values after induction for 48 h. Mean values in the same line (row) not sharing the same letter are significantly different by ANOVA Tukey test (*p* < 0.05).

## Data Availability

Data are contained within the article and Supplementary Materials.

## Supplementary Materials

The following supporting information can be downloaded at: https://www.mdpi.com/article/10.3390/molecules29163887/s1, Figures S1–S7: HPLC profiles of samples *Ps2*–*Ps8*. Figures S8–S15: Graphs of the cytotoxic effect of samples *Ps1*–*Ps8*. Figures S16–S19: Graphs of the cytotoxic effect of compounds **3**–**6**.
