# Supplementary material for: Chemical Characterization of Pruning Wood Extracts from Six Japanese Plum (Prunus salicina Lindl.) Cultivars and Their Antitumor Activity
Source: Molecules. 2024 Aug 16;29(16):3887. doi: 10.3390/molecules29163887 (PMC11357068; doi:10.3390/molecules29163887)
Supplement: Supplementary file 1 [file molecules-29-03887-s001.zip › molecules-3104395-supplementary.pdf]

Supplementary Materials

Chemical Characterization of Pruning Wood Extracts from Six Japanese Plum (*Prunus salicina* Lindl.) Cultivars and their Antitumor Activity

Juan Ortega-Vidal [1], Nuria Mut-Salud [2], José M. de la Torre [1], Joaquín Altarejos [1,*] and Sofía Salido [1]

[1] Department of Inorganic and Organic Chemistry, Faculty of Experimental Sciences, University of Jaén, Campus of International Excellence in Agri-Food (ceiA3), 23071 Jaén, Spain; jov00007@red.ujaen.es (J.O.-V.); jmtorre@ujaen.es (J.M.T.); ssalido@ujaen.es (S.S.)
[2] Institute of Biopathology and Regenerative Medicine, University of Granada, 18071 Granada, Spain; nmutsalud@gmail.com (N.M.-S)
* Correspondence: jaltare@ujaen.es (J.A.)

Table of contents

Figure S1.

HPLC profile of sample *Ps2*

Figure S2.

HPLC profile of sample *Ps3*

Figure S3.

HPLC profile of sample *Ps4*

Figure S4.

HPLC profile of sample *Ps5*

Figure S5.

HPLC profile of sample *Ps6*

Figure S6.

HPLC profile of sample *Ps7*

Figure S7.

HPLC profile of sample *Ps8*

Figure S8.

Graph of the cytotoxic effect of sample *Ps1* in MCF-7 cells.

Figure S9.

Graph of the cytotoxic effect of sample *Ps2* in MCF-7 cells.

Figure S10.

Graph of the cytotoxic effect of sample *Ps3* in MCF-7 cells.

Figure S11.

Graph of the cytotoxic effect of sample *Ps4* in MCF-7 cells.

Figure S12.

Graph of the cytotoxic effect of sample *Ps5* in MCF-7 cells.

Figure S13.

Graph of the cytotoxic effect of sample *Ps6* in MCF-7 cells.

Figure S14.

Graph of the cytotoxic effect of sample *Ps7* in MCF-7 cells.

Figure S15.

Graph of the cytotoxic effect of sample *Ps8* in MCF-7 cells.

Figure S16.

Graph of the cytotoxic effect of compound **3** in MCF-7 cells.

Figure S17.

Graph of the cytotoxic effect of compound **4** in MCF-7 cells.

Figure S18.

Graph of the cytotoxic effect of compound **5** in MCF-7 cells.

Figure S19.

Graph of the cytotoxic effect of compound **6** in MCF-7 cells.

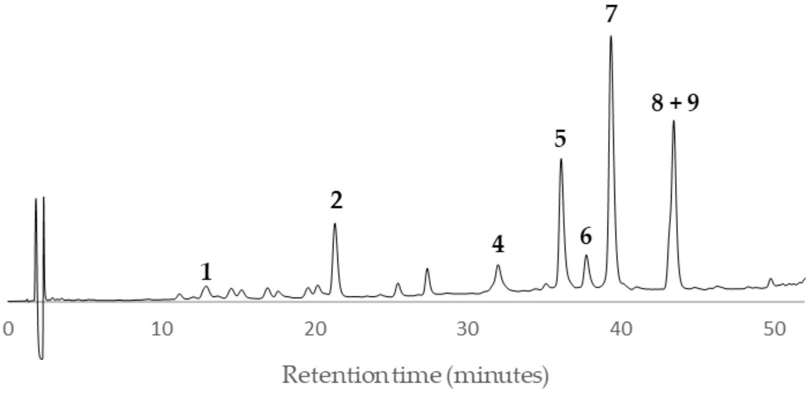

Figure S1. HPLC chromatogram of *Prunus salicina* Lindl. cv. 'Angeleno' wood EtOAc extract (sample *Ps2*) at 230 nm.

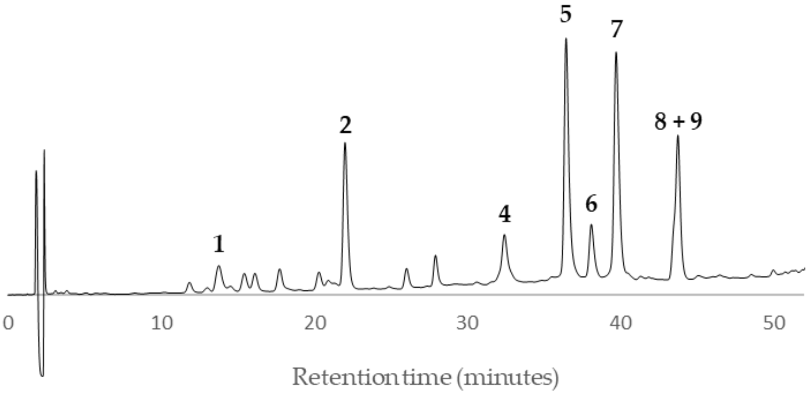

Figure S2. HPLC chromatogram of *Prunus salicina* Lindl cv. 'Angeleno' wood EtOAc extract (sample *Ps3*) at 230 nm.

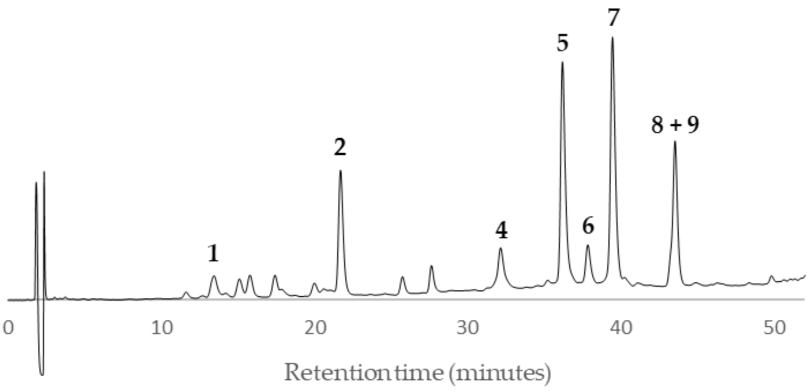

Figure S3. HPLC chromatogram of *Prunus salicina* Lindl. cv. 'Angeleno' wood EtOAc extract (sample *Ps4*) at 230 nm.

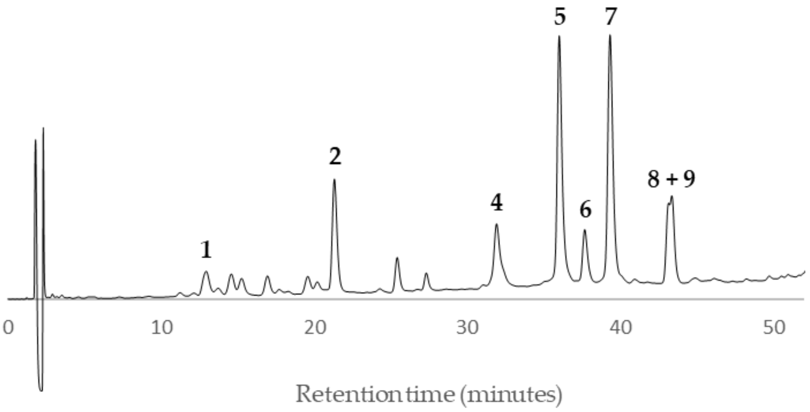

Figure S4. HPLC chromatogram of *Prunus salicina* Lindl. cv. 'Fortune' wood EtOAc extract (sample *Ps5*) at 230 nm.

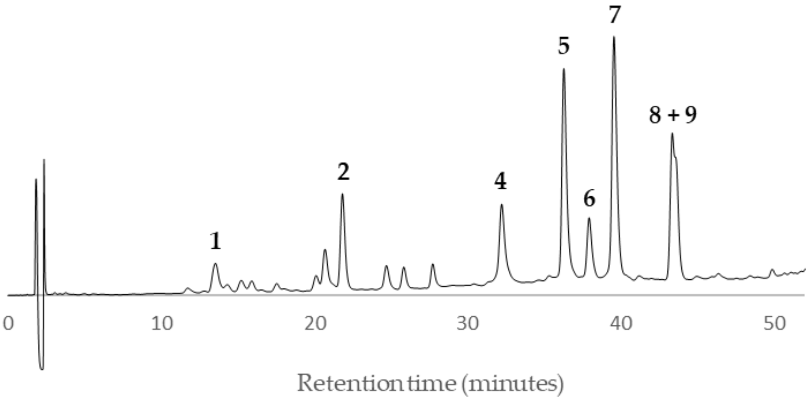

Figure S5. HPLC chromatogram of *Prunus salicina* Lindl. cv. 'Red Beaut' wood EtOAc extract (sample *Ps6*) at 230 nm.

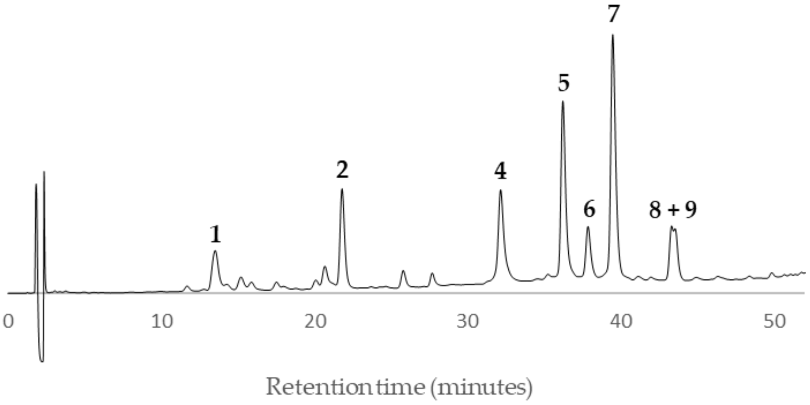

Figure S6. HPLC chromatogram of *Prunus salicina* Lindl. cv. 'Souvenir' wood EtOAc extract (sample *Ps7*) at 230 nm.

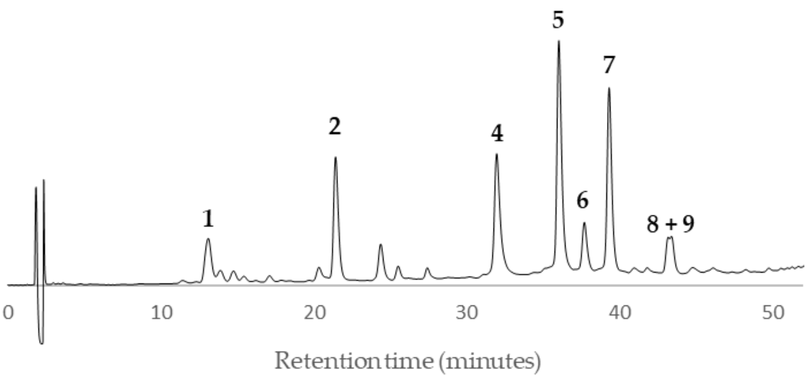

Figure S7. HPLC chromatogram of *Prunus salicina* Lindl. cv. 'Showtime' wood EtOAc extract (sample *Ps8*) at 230 nm.

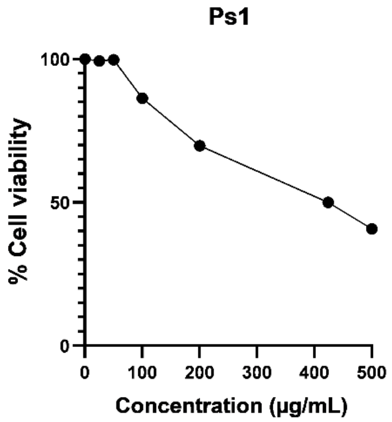

Figure S8. Cytotoxic effect of *Prunus salicina* Lindl. cv. 'Songold' wood EtOAc extract (sample *Ps1*) in MCF-7 cells.

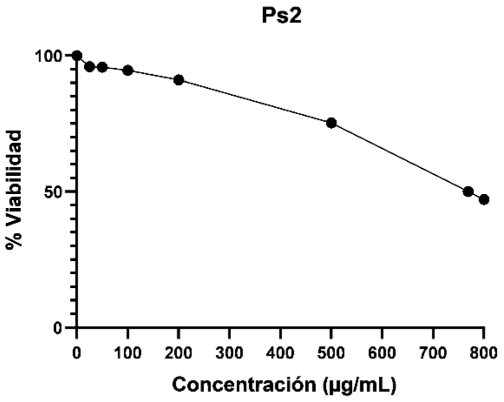

Figure S9. Cytotoxic effect of *Prunus salicina* Lindl. cv. 'Angeleno' wood EtOAc extract (sample *Ps2*) in MCF-7 cells.

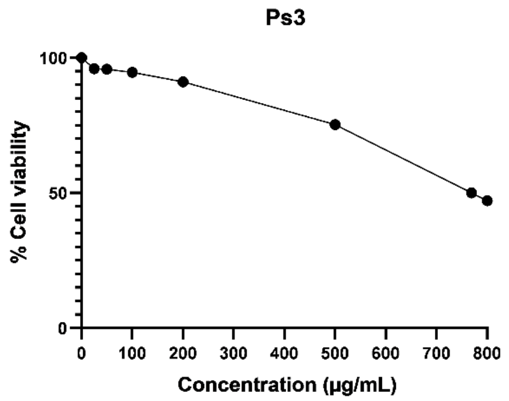

Figure S10. Cytotoxic effect of *Prunus salicina* Lindl. cv. 'Angeleno' wood EtOAc extract (sample *Ps3*) in MCF-7 cells.

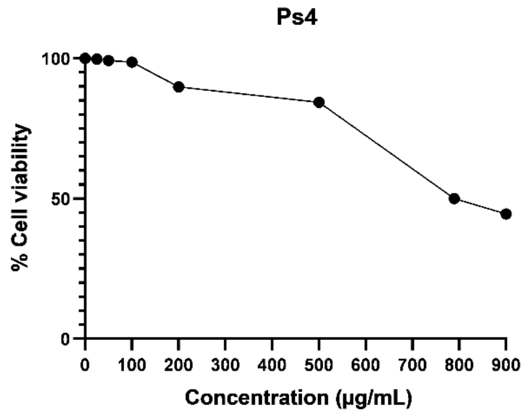

Figure S11. Cytotoxic effect of *Prunus salicina* Lindl. cv. 'Angeleno' wood EtOAc extract (sample *Ps4*) in MCF-7 cells.

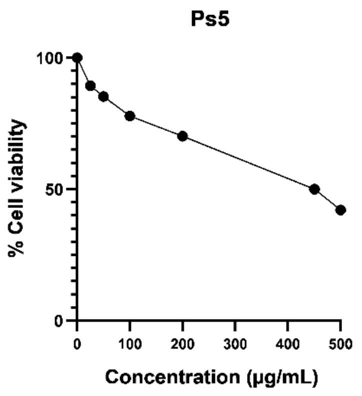

Figure S12. Cytotoxic effect of *Prunus salicina* Lindl. cv. 'Fortune' wood EtOAc extract (sample *Ps5*) in MCF-7 cells.

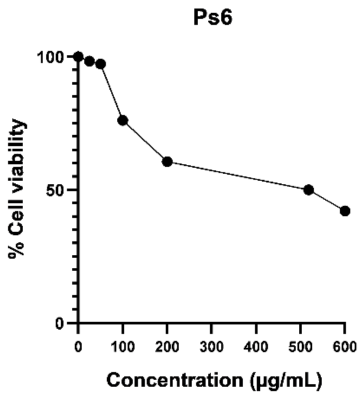

Figure S13. Cytotoxic effect of *Prunus salicina* Lindl. cv. 'Red Beaut' wood EtOAc extract (sample *Ps6*) in MCF-7 cells.

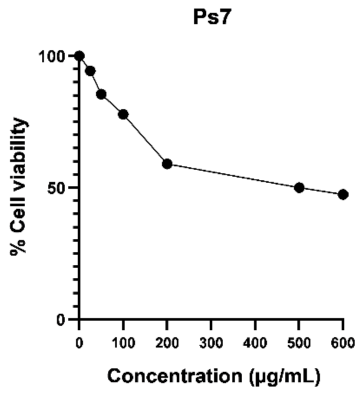

Figure S14. Cytotoxic effect of *Prunus salicina* Lindl. cv. 'Souvenir' wood EtOAc extract (sample *Ps7*) in MCF-7 cells.

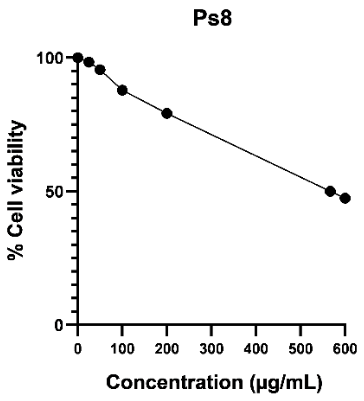

Figure S15. Cytotoxic effect of *Prunus salicina* Lindl. cv. 'Showtime' wood EtOAc extract (sample *Ps8*) in MCF-7 cells.

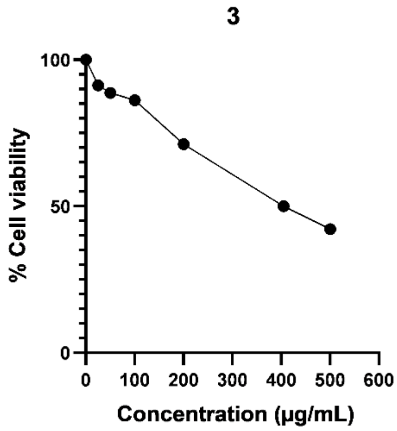

Figure S16. Cytotoxic effect of (–)-annphenone (**3**) in MCF-7 cells.

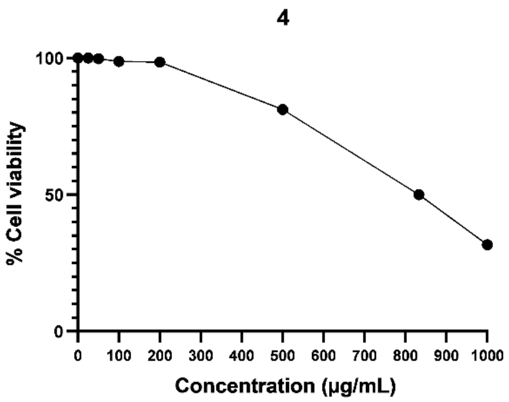

Figure S17. Cytotoxic effect of (–)-*ent*-epicatechin-(2*α*→*O*→7,4*α*→8)-catechin (**4**) in MCF-7 cells.

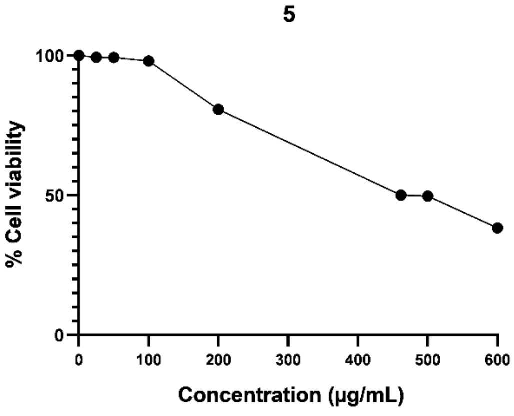

Figure S18. Cytotoxic effect of (–)-*ent*-epicatechin-(2*α*→*O*→7,4*α*→8)-epicatechin (**5**) in MCF-7 cells.

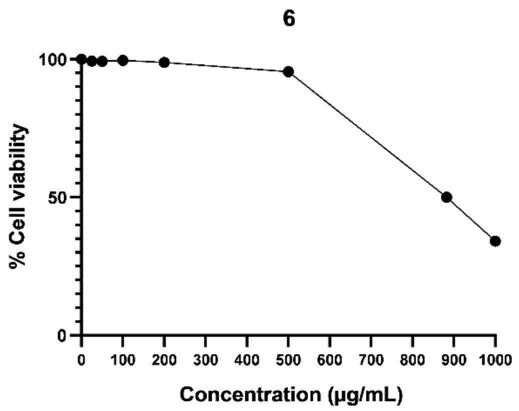

Figure S19. Cytotoxic effect of (–)-*ent*-epiafzelechin-(2α→*O*→7,4α→8)-catechin (**6**) in MCF-7 cells.
